# Neoadjuvant Chemotherapy in Advanced Stage Endometrial Cancer: A Systematic Review and Meta-Analysis

**DOI:** 10.3390/medicina62010130

**Published:** 2026-01-08

**Authors:** Maria Fanaki, Dimitrios Haidopoulos, Antonia Varthaliti, Dimitrios Efthimios Vlachos, Georgios Daskalakis, Nikolaos Thomakos, Vasilios Pergialiotis

**Affiliations:** First Department of Obstetrics and Gynecology, Division of Gynecologic Oncology, “Alexandra” General Hospital, National and Kapodistrian University of Athens, 115 28 Athens, Greece; dimitrioshaidopoulos@gmail.com (D.H.); antonia.varthaliti@hotmail.com (A.V.); vlachos.dg@gmail.com (D.E.V.); gdaskalakis@yahoo.com (G.D.); thomakir@hotmail.com (N.T.)

**Keywords:** endometrial cancer, uterine cancer, neoadjuvant chemotherapy

## Abstract

*Background and Objectives*: Endometrial cancer is the most common gynecological malignancy in developed countries and is becoming increasingly prevalent. Early diagnosis and treatment may lead to lower rates of morbidity and mortality. The aim of the present meta-analysis is to investigate whether neoadjuvant chemotherapy (NACT) can enhance resectability, reduce tumor burden, and ultimately improve survival rates compared to primary surgery in patients with advanced endometrial cancer. *Materials and Methods*: All studies that examined the impact of NACT on survival outcomes of patients with advanced endometrial cancer were eligible for inclusion, including randomized and non-randomized interventional studies. Studies were identified by searching MEDLINE (1945–2024), Scopus (1941–2024), Google Scholar (2004–2024) and ClinicalTrials.gov (2000–2024). Data was selected and extracted by two reviewers based on the PRISMA guidelines. *Results*: Five retrospective studies with a cumulative total of 8658 patients were included. No statistically significant difference in overall survival was observed between patients who received NACT and those who underwent primary surgery (HR 0.91, 95% CI 0.79–1.04). NACT was associated with some perioperative advantages, though these did not translate into a survival benefit. *Conclusions*: The currently available evidence, which is limited to retrospective studies with significant heterogeneity, suggests that NACT does not confer a survival advantage over primary debulking surgery in advanced endometrial cancer. These findings should be considered hypothesis-generating, underscoring the need for prospective trials. NACT may still be a reasonable option for selected subgroups, such as frail patients, those with extensive peritoneal disease, or cases in which complete cytoreduction is unlikely with upfront surgery.

## 1. Introduction

Endometrial cancer is a major and growing global health concern. According to recent GLOBOCAN estimates, there were approximately 417,000 new cases of endometrial cancer worldwide in 2020, representing about 2.2% of all new cancer diagnoses, and nearly 97,400 deaths were attributed to this disease in the same year. Approximately 90,000 people die from endometrial cancer worldwide each year. The American Cancer Society reports that the 5-year survival rate for early-stage endometrial cancer can exceed 90%. Moreover, global cancer surveillance data indicate that the overall cancer burden continues to rise, with nearly 20 million new cancer cases and 9.7 million cancer deaths reported in 2022, and future projections suggesting substantial increases in cancer incidence by 2050 due to demographic and lifestyle changes [1]. However, if the cancer has spread, the prognosis becomes poorer, with the 5-year survival rate for stage IV endometrial cancer falling to 15–20%. The standard treatment for advanced endometrial cancer typically involves primary debulking surgery followed by adjuvant therapy. However, for patients with unresectable disease or poor performance status, neoadjuvant chemotherapy (NACT) has been suggested as a potential alternative to improve surgical outcomes and overall survival.

It is well established that the use of NACT followed by interval debulking surgery has become a standard therapeutic option for advanced ovarian cancer [2]. In particular, the survival rates for women with ovarian cancer who undergo primary debulking surgery followed by adjuvant therapy or neoadjuvant chemotherapy are approximately comparable [3]. Given that high-grade serous ovarian cancer demonstrates similar intraperitoneal spread patterns and shares molecular phenotypes such as p53 mutations, BRCA pathway disruptions, and a high rate of genomic instability with endometrial cancer, particularly serous uterine cancer, it is critical to examine the clinical evidence for employing either approach in such patients too [4,5,6]. However, important biological differences exist endometrial cancer—especially in advanced and recurrent stages—often demonstrates distinct chemoresistance mechanisms and only modest responsiveness to first-line chemotherapy [7]. These differences may limit the direct translatability of the ovarian cancer paradigm to endometrial cancer and highlight the need for disease-specific evidence before adopting NACT broadly.

Neoadjuvant therapy has been proposed as a strategy for reducing the morbidity of debulking surgery while improving the feasibility of complete debulking, improving survival outcomes and reducing recurrence rates [8,9]. However, other researchers contradict these findings, suggesting that survival rates may be poorer when neoadjuvant chemotherapy is implemented [10]. While both strategies—primary surgery followed by adjuvant therapy and NACT followed by interval debulking—are currently utilized in clinical practice, there is no consensus on which strategy produces better outcomes in terms of mortality, morbidity, and quality of life.

In the present systematic review and meta-analysis, we evaluated the efficacy and safety of neoadjuvant chemotherapy in patients with advanced endometrial cancer by synthesizing data from randomized and non-randomized studies. The aim of our study is to investigate whether NACT can enhance resectability, reduce tumor burden, and ultimately improve survival rates compared to primary surgery in patients with advanced endometrial cancer.

## 2. Methods

This systematic review was prospectively registered in the International Prospective Register of Systematic Reviews (PROSPERO; registration number: CRD42024593209) prior to study initiation and was performed in accordance with the Preferred Reporting Items for Systematic Reviews and Meta-Analyses (PRISMA) guidelines [11]. As the analysis was based exclusively on data extracted from previously published studies, neither institutional review board approval nor informed patient consent was required

### 2.1. Eligibility Criteria, Information Sources, Search Strategy

Study eligibility criteria were established in advance. Randomized and non-randomized interventional studies investigating the association between neoadjuvant chemotherapy and survival outcomes in advanced endometrial cancer were eligible for inclusion, irrespective of histological subtype. Subgroup analyses based on molecular profile and histological classification (type I/type II) were planned when adequate data were available. Case reports, laboratory studies, and conference proceedings were excluded.

**Figure 1:**
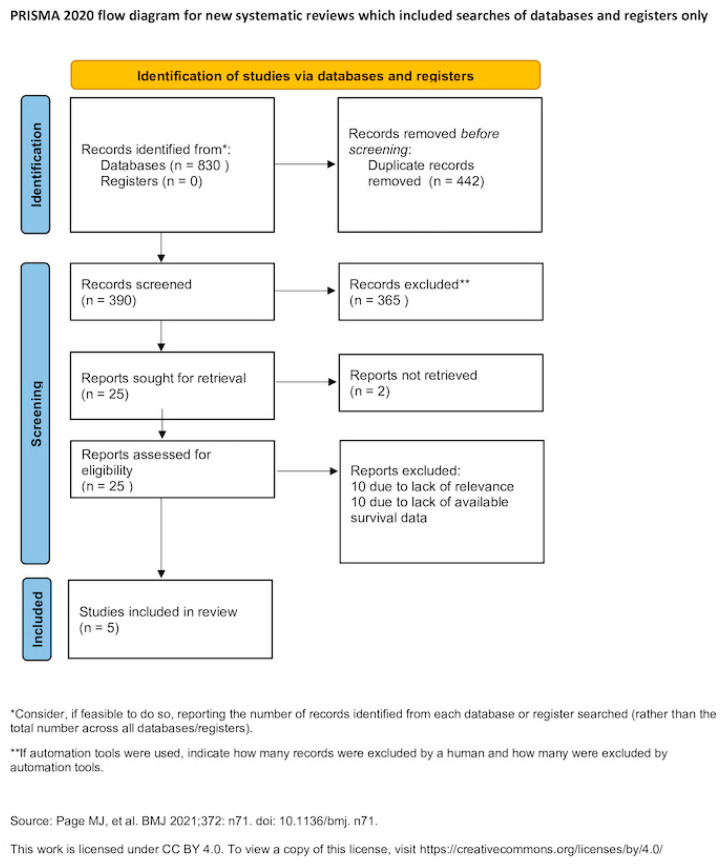
Search plot diagram [12].

A comprehensive literature search was conducted across multiple electronic databases, including MEDLINE (1945–2024), Scopus (1941–2024), Google Scholar (2004–2024), and ClinicalTrials.gov (2000–2024). In addition, the reference lists of all eligible studies, as well as those of relevant systematic reviews and meta-analyses, were manually reviewed to identify further studies not captured by the initial search strategy. No restrictions were imposed on publication date. The search was limited to studies published in languages using the Latin alphabet; articles published in languages other than English, French, German, Italian, or Spanish were translated using online translation tools, as specified a priori. The final search was completed on 30 September 2024. Search terms included “endometrial cancer,” “uterine cancer,” and “neoadjuvant chemotherapy,” and the full search strategy is illustrated in Figure 1.

### 2.2. Study Selection

The study selection process comprised three stages. Duplicate records were first eliminated, followed by independent screening of titles and abstracts by two authors (M.F. and V.P.). Full-text assessment was then conducted for studies meeting initial eligibility criteria, with discrepancies resolved by consensus.

### 2.3. Data Extraction

The outcomes evaluated in this review were established before data collection commenced. Information was extracted using an adapted version of the Cochrane data extraction framework for interventional studies. The primary endpoint assessed was the association between neoadjuvant chemotherapy and survival outcomes, namely overall and recurrence-free survival, in patients with advanced endometrial cancer. Secondary endpoints encompassed postoperative residual disease, perioperative adverse events, and measures of surgical complexity.

### 2.4. Assessment of Risk of Bias

The methodological quality of the included observational studies was independently evaluated by two reviewers (M.F. and V.P.) using the Newcastle–Ottawa Scale (NOS). This instrument assesses risk of bias across three domains: selection of study groups (maximum of 4 points), comparability of cohorts (maximum of 2 points, awarded for comparability with respect to histological subtype and disease stage), and assessment of outcomes (maximum of 3 points), with adequate outcome assessment defined as a median follow-up of at least three years, as reported by the authors or inferred from an interval of ≥3 years between final patient recruitment and publication [13].

### 2.5. Data Synthesis

Quantitative analysis was performed, provided that published data in the field provided the relevant information and that outcome reporting measures will be sufficiently homogenous. In cases where these criteria were not met, a descriptive synthesis was performed, accompanied by an analysis of factors contributing to substantial heterogeneity among the included studies to inform future research. Meta-analysis was performed with RStudio using the meta function (RStudio Team, 2015: Integrated Development for R. RStudio, Inc., Boston, MA, USA. URL http://www.rstudio.com/).

Given the substantial methodological heterogeneity among the included studies (Table 1), statistical heterogeneity was not used to guide model selection, as the assumption of a common underlying effect size was not considered appropriate. To reduce the influence of potential confounding factors—such as disease stage, tumor grade, histological subtype, and patient comorbidities—effect estimates derived from multivariable analyses were preferentially extracted over unadjusted univariate results. All estimates are presented with 95% confidence intervals. Pooled odds ratios and mean differences were calculated using the Hartung–Knapp–Sidik–Jonkman approach rather than the conventional DerSimonian–Laird random-effects model, based on evidence supporting its superior performance in the presence of between-study heterogeneity and unequal sample sizes [14].

During the design of this systematic review, we selected the Egger’s test as a statistical method to evaluate the possibility of publication bias. This method represents a linear regression analysis that examines the intervention effect estimates and their standard errors which are weighted by their inverse variance [20]. It is considered important only when substantial evidence is present and a prerequisite of at least 10 studies was predefined as a minimum threshold per investigated outcome to ensure appropriate credibility of retrieved findings [21].

To assess the potential impact of small-study effects on the pooled estimates, Rücker’s limit meta-analysis was conducted, allowing evaluation of whether effect sizes varied according to study precision. In addition, p-curve analysis was performed to examine the evidential value of the aggregated findings and to explore the possibility of selective reporting or data manipulation.

Prediction intervals were additionally calculated using the *meta* package in RStudio (RStudio Team, 2015: Integrated Development for R. RStudio, Inc., Boston, MA, USA. URL http://www.rstudio.com/). to estimate the range of effects that may be observed in future studies. Unlike confidence intervals, prediction intervals incorporate between-study variability and therefore provide an expression of heterogeneity on the same scale as the outcome of interest.

## 3. Results

Overall, 25 articles were considered for potential inclusion in the present systematic review [9,10,11,15,16,18,19,22,23,24,25,26,27,28,29,30,31,32,33,34,35,36,37]. Of those, 20 articles were excluded due to absence of relevance or lack of appropriate outcome reporting [9,11,22,23,24,25,26,27,28,29,30,31,32,33,34,35,36,37]. The remaining 5 articles that were included in the present meta-analysis recruited 8658 patients. The methodological characteristics and heterogeneity of included studies is summarized in Table 1. Discrepancies were noted among the histological types of tumors that were selected for analysis as well as in the stage of disease at diagnosis, and comorbidities of the patients included. For this reason, survival outcomes were retrieved from multivariate Cox-regression analyses that considered variables that were found statistically significant in the univariate analysis, except for one study that provided results from univariate Kaplan–Meier analysis only [15], and one study demonstrated outcomes from a propensity score–balanced cohort [10]. Patient and tumor characteristics are presented in Table 2. Notable disparities were observed in the prevalence of adverse prognostic factors among patients receiving neoadjuvant chemotherapy. Researchers specifically noted a heightened incidence of advanced stage disease and endometrioid type uterine cancer in patients undergoing neoadjuvant chemotherapy, which subsequently led to disparities in survival outcomes when compared to patients receiving primary cytoreduction.

The assessment of the quality of retrieved studies revealed that most of them had rigorous methodology that precluded the possibility of selection bias (Table 3). In terms of comparability, most studies revealed non-significant differences in the histology or the stage of tumors among patients that received that compared to patients underwent primary debulking surgery.

**Figure 2:**
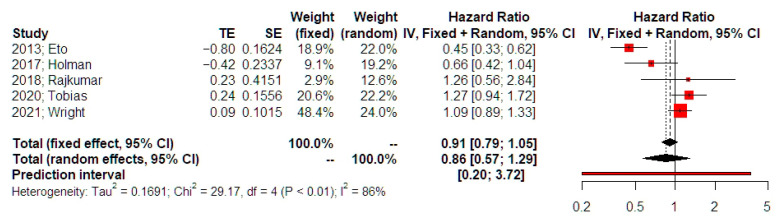
Hazards ratio of overall survival. Forest plot analysis: Vertical line = “no difference” point between the two groups. Red squares = hazard ratios of included studies; Horizontal black lines = 95% CI of included studies; Diamond = pooled hazard ratios retrieved from the outcomes of the meta-analysis and 95% CI for all studies; Horizontal red line = prediction intervals. The weight of included studies is depicted for fixed and random effects model separately [15,16,17,18,19].

The meta-analysis of data from 5 studies did not reveal a significant difference in overall survival in patients with advanced stages of endometrial cancer that received neoadjuvant chemotherapy comparing to those who had primary surgery (HR 0.91, 95% CI 0.79, 1.04, Figure 2). Substantial statistical heterogeneity was noted (I-square test = 86%). Prediction intervals indicated that future studies might corroborate the statistical significance that is evident in the present meta-analysis. Sensitivity analysis indicated that the possibility of small study effects was minimal (*p* = 0.211). The adjusted estimate for overall survival was not statistically strong HR = 0.79 (95% 0.555, 1.125). The limited number of eligible papers also precluded Egger’s test and Funnel plot analyses. Influential (leave-one-out) analysis (random effects model) did not indicate the presence of a study that could significantly deviate the statistical significance of the primary analysis (Appendix S1).

## 4. Discussion

This meta-analysis aimed to compare the survival outcomes of neoadjuvant chemotherapy (NACT) versus primary debulking surgery (PDS) in the treatment of advanced-stage endometrial cancer. The analysis showed that although NACT is considered to have some benefits regarding perioperative morbidity, it did not offer better or even equal survival benefits compared to PDS. Our findings suggest that while NACT may confer some perioperative advantages, such as reduced operative morbidity, it did not demonstrate a statistically significant survival advantage over PDS. The pooled analysis (HR 0.91, 95% CI 0.79–1.04) indicates no significant difference in overall survival between the two approaches. Although some individual studies reported less favorable outcomes for NACT, these findings should be interpreted with caution given the retrospective design and heterogeneity of included cohorts. Thus, the results are best considered hypothesis-generating rather than definitive.

Stage IVB endometrial cancer, as defined in the 2023 FIGO staging system, includes a diverse group of patients with varying disease distribution, such as intra- and extra-abdominal metastasis [17]. The presence of peritoneal carcinomatosis and/or distant or parenchymal metastasis complicates the development of definitive recommendations for optimal primary treatment and adjuvant therapy. Current guidelines recommend primary cytoreductive surgery when feasible, but there are no strict indications for adjuvant treatment. The use of NACT is supported by growing evidence from ovarian cancer, where it has shown promising results. Three randomized trials showed that interval debulking surgery yields comparable survival outcomes to primary debulking surgery in ovarian cancer patients [38,39,40]. However, unlike ovarian cancer, endometrial cancer often displays distinct biological behavior, including relative chemoresistance, particularly in advanced stages, which may limit the effectiveness of NACT as a primary strategy [4,41].

Evidence from retrospective studies have been inconclusive regarding the survival benefits of NACT compared to PDS. Some studies indicate no significant improvement in overall survival with NACT, while others suggest potential benefits in specific patient subsets. In a retrospective small cohort that included 39 patients with advanced endometrial cancer, and they observed that patients that had a positive response to NACT, had significantly prolonged overall survival than those who did not respond favorably [42]. The survival was better especially for the patients that, underwent IDS after NACT compared to patients that did not undergo surgery. Moreover, Khouri, et al. observed that patients who underwent interval cytoreductive surgery after neoadjuvant chemotherapy had longer progression-free survival rate (12.53 vs. 5 months, *p* = 0.001) and longer overall survival rate (25 vs. 8 months, *p* = 0.002) in comparison with patients who have not undergone surgery [35].

On the other hand, another retrospective study found that patients treated with NACT had a considerably poorer overall survival of 22.89 months compared to those treated by primary surgery, who had an overall survival of 56.30 months [36]. Tayeb et al. conducted a retrospective study which included 44 patients with stage III–IV uterine serous carcinoma and revealed no significant difference regarding the overall and disease-free survival. Furthermore, more patients in the NACT group achieved complete cytoreduction than those who underwent PDS (70% vs. 32%). Furthermore, 18% of PDS patients had persistent tumors, which required prolonged surgery and hospitalization [11]. In alignment with this, a retrospective study with total 5505 patients with stage IVB EC showed similar overall survival among the NACT group and the PDS group, 25 months and 26 months, respectively.

Furthermore, it was observed that any perioperative advantages including shorter operative time, lower transfusion rate, and shorter length of stay offered by NACT did not translate into long-term survival benefits [23,43]. Several factors may account for this lack of survival advantage for EC patients who received NACT. First, NACT may delay the timing of surgery, allowing disease progression in some patients before an optimal cytoreduction can be achieved. Unlike ovarian cancer, where NACT has shown to be beneficial in certain cases, endometrial cancer may have a different biological behavior and response to chemotherapy. The chemoresistance of endometrial cancer, particularly in advanced stages, could undermine the effectiveness of NACT as an initial treatment strategy. It is well established that advanced and recurrent endometrial carcinomas remain a challenging group of tumors that are only modestly responsive to first-line chemotherapy and demonstrate high rates of multifactorial chemotherapy resistance [10].

Second, the extent of disease in patients receiving NACT may still be suboptimal at the time of surgery, leading to residual disease that impacts survival. In our analysis, it was observed that patients treated by primary surgery underwent more extended procedures and presented more often postoperative residual disease compared to patients that received neoadjuvant chemotherapy [16,18]. PDS aims for maximal cytoreduction, which has been repeatedly associated with improved survival in gynecologic cancers [44]. In endometrial cancer, optimal cytoreduction, with minimal residual disease, remains a crucial prognostic factor for survival. The inability to achieve similar cytoreductive outcomes after NACT might explain the poorer survival outcomes in this group [45]. The results of a recent meta-analysis show that among patients with advanced-stage endometrial cancer undergoing primary cytoreductive surgery, just over half achieved no gross residual disease and three-quarters achieved <1 cm residual disease, with lower rates in stage IV compared to stage III–IV disease, and any residual disease was consistently associated with significantly worse progression-free and overall survival [46].

This meta-analysis has several limitations. Only five retrospective studies were included, encompassing heterogeneous populations with variations in staging, histology, surgical approach, and chemotherapy regimens (Supplementary Table S1). Such heterogeneity limits comparability and contributes to high statistical variance. Additionally, the retrospective design introduces inherent risks of selection bias and unmeasured confounding. In addition, racial and ethnic characteristics were variably reported across the included studies. Detailed racial data were available in three population-based studies conducted in the United States, whereas ethnicity was not reported or incompletely documented in the remaining studies. These limitations underscore the need for prospective, ideally randomized, studies to establish more definitive evidence on the role of NACT in advanced endometrial cancer. NACT may still be a reasonable option for selected subgroups, such as frail patients, those with extensive peritoneal disease, or cases in which complete cytoreduction is unlikely with upfront surgery.

## 5. Conclusions

The results of this meta-analysis suggest that NACT does not provide significantly improved survival outcomes in advanced-stage endometrial cancer patients. Therefore, primary surgery may be considered with the remaining question of the impact of suboptimal debulking on survival outcomes. Therefore, it seems to be prudent to support that NACT may still have a role in selected populations when complete tumor resection is considered impossible. Nonetheless, NACT may still be appropriate for selected patient groups, particularly when complete tumor removal is not achievable. Additionally, frail patients or those with significant comorbidities may benefit from NACT, as aggressive surgical approaches in these populations are often linked to high morbidity and mortality.

## Figures and Tables

**Table 1:** Study characteristics.

Study; Year	Country	Study	Patients	Inclusion Criteria	Exclusion Criteria
Eto; 2013 [15]	Japan	Retrospective	426	Patients diagnosed with clinical or surgical FIGO 1988 stage IVb endometrial cancer from 1996 to 2005.	Patients with sarcoma were excluded.
Holman; 2017 [16]	USA	Retrospective	260	Patients with stage III or IV endometrial cancer as defined by FIGO 2009 between March 1993 and January 2012. Any tumor with more than 5% serous component was included.	Women with inadequate data in the medical record, synchronous primary tumors, or carcinosarcoma were excluded.
Rajkumar; 2019 [17]	England	Retrospective	45	Patients diagnosed with macroscopic or radiological Stage IIIC/IV Endometrial carcinoma (FIGO 2009) between January 2010 and December 2016.	Patients who received palliative chemotherapy or hormones (due to extensive disseminated disease or poor performance status) and those with sarcomatous histology were excluded.
Tobias; 2020 [18]	USA	Cohort	4890	Patients with stage IV malignant uterine cancer diagnosed as their only or first cancer with pathological confirmation from 1 January 2010, to 31 December 2015.	Patients older than 70 years, with Charlson comorbidity score greater than 0, who had neoadjuvant or intraoperative radiation therapy, for whom administration of chemotherapy was unknown or the time of chemotherapy administration was uncertain, and who had chemotherapy and surgery (hysterectomy or exenteration) initiated on the same day or more than 90 days after cancer diagnosis. Comorbidity scores greater than 0 were excluded.
Wright; 2021 [19]	Population-based analysis	USA	3037	Patients older than 65 years with stage IV uterine cancer diagnosed as the first or only cancer confirmed with positive histology from 2000 to 2015.	Patients who were diagnosed from autopsy or death certificate, had a date of death inconsistent between SEER and Medicare databases, or with no follow-up data were excluded.

**Table 2:** Patient and tumor characteristics.

Study; Year	Chemotherapy Regimens
Eto; 2013 [15]	Taxanes/Platinum: 78/125 Doxorubicin/Platinum 38/125 Other: 9/125
Holman; 2017 [16]	NA
Rajkumar; 2019 [17]	Carboplatin/Taxol: 14/17 Cisplatin/Doxorubicin: 2/17 Capecitabine: 1/17
Tobias; 2020 [18]	NA
Wright; 2021 [19]	NA

NA: Not Available.

**Table 3:** Newcastle–Ottawa scale assessment.

Study	Selection				Comparability	Outcome		
	Adequacy of Case Definition	Representativeness of Cases	Selection of Controls	Definition of Controls	Comparability (Histology/Stage)	Assessment of Outcome	Adequate Follow-Up Period	Adequate Follow-Up
Eto; 2013 [15]	√	√	√	√	√- *	√	√	√
Holman; 2017 [16]	√	√	√	√	√√ *	√	√	√
Rajkumar; 2019 [17]	√	√	√	√	√√ *	√	√	√
Tobias; 2020 [18]	√	√	√	√	√√ *	√	√	√
Wright; 2021 [19]	√	√	√	√	√√ *	√	√	√

* multivariate analysis with the Cox-regression model accounting for factors that significantly differed among patients with advanced staged of endometrial cancer who received NACT. √√ it means the study received full marks for the Comparability domain.√>: it means partyial comparability.

## Data Availability

Data available upon reasonable request.

## Supplementary Materials

The following supporting information can be downloaded at https://www.mdpi.com/article/10.3390/medicina62010130/s1, Supplementary Table S1. Chemotherapy Regimens. Appendix S1.
