# Supplementary material for: Neoadjuvant Chemotherapy in Advanced Stage Endometrial Cancer: A Systematic Review and Meta-Analysis
Source: Medicina (Kaunas). 2026 Jan 8;62(1):130. doi: 10.3390/medicina62010130 (PMC12843081; doi:10.3390/medicina62010130)
Supplement: Supplementary file 1 [file medicina-62-00130-s001.zip › Appendix S1.pdf]

Appendix

```
Identified outliers (random-effects model)
------------------------------------------
""

Results with outliers removed
-----------------------------
                   HR           95%-CI      %W(fixed)  %W(random)
Exclude
2013; Eto      0.4490 [0.3266; 0.6173]      18.9       22.0
2017; Holman   0.6600 [0.4175; 1.0435]       9.1       19.2
2018; Rajkumar 1.2600 [0.5585; 2.8425]       2.9       12.6
2020; Tobias   1.2700 [0.9362; 1.7228]      20.6       22.2
2021; Wright   1.0900 [0.8934; 1.3299]      48.4       24.0

Number of studies combined: k = 5

                         HR            95%-CI     z p-value
Fixed effect model   0.9123 [0.7944; 1.0478] -1.30  0.1940
Random effects model 0.8581 [0.5716; 1.2883] -0.74  0.4604

Quantifying heterogeneity:
 tau^2 = 0.1691 [0.0408; 1.6944]; tau = 0.4112 [0.2020; 1.3017]
 I^2 = 86.3% [70.1%; 93.7%]; H = 2.70 [1.83; 3.99]

Test of heterogeneity:
     Q d.f.  p-value
 29.17    4 < 0.0001

Details on meta-analytical method:
- Inverse variance method
- Sidik-Jonkman estimator for tau^2
- Q-profile method for confidence interval of tau^2 and tau



Influential (leave-one-out) analysis (Random effects model)

                              HR            95%-CI p-value   tau^2   I^2
Omitting 2013; Eto        1.0389 [0.7688; 1.4039]  0.8040  0.0532  47.1%
Omitting 2017; Holman     0.9160 [0.5598; 1.4987]  0.7267  0.2070  88.9%
Omitting 2018; Rajkumar   0.8104 [0.5089; 1.2907]  0.3760  0.1977  89.5%
Omitting 2020; Tobias     0.7670 [0.4846; 1.2140]  0.2575  0.1676  87.2%
Omitting 2021; Wright     0.7993 [0.4821; 1.3252]  0.3852  0.2077  87.1%

Pooled estimate           0.8581 [0.5716; 1.2883]  0.4604  0.1691  86.3%

Details on meta-analytical method:
- Inverse variance method
- Sidik-Jonkman estimator for tau^2
```

```
Analysis for small study effects
--------------------------------

                    HR           95%-CI       HR           95%-CI
2013; Eto       0.4490 [0.3266; 0.6173]    0.4456 [0.3241; 0.6125]
2017; Holman    0.6600 [0.4175; 1.0435]    0.6186 [0.3913; 0.9781]
2018; Rajkumar  1.2600 [0.5585; 2.8425]    0.8983 [0.3982; 2.0264]
2020; Tobias    1.2700 [0.9362; 1.7228]    1.1789 [0.8690; 1.5992]
2021; Wright    1.0900 [0.8934; 1.3299]    1.0588 [0.8678; 1.2918]

Result of limit meta-analysis:

 Random effects model     HR           95%-CI     z   pval
    Adjusted estimate 0.7907 [0.5553; 1.1259] -1.30 0.1928
  Unadjusted estimate 0.8581 [0.5716; 1.2883] -0.74 0.4604

Quantifying heterogeneity:
tau^2 = 0.1691; I^2 = 86.3% [70.1%; 93.7%]; G^2 = 96.2%

Test of heterogeneity:
     Q d.f.  p-value
 29.17    4 < 0.0001

Test of small-study effects:
  Q-Q' d.f.  p-value
  1.56    1   0.2117

Test of residual heterogeneity beyond small-study effects:
    Q' d.f.  p-value
 27.61    3 < 0.0001
```
