# Supplementary material for: Neoadjuvant Chemotherapy in Advanced Stage Endometrial Cancer: A Systematic Review and Meta-Analysis
Source: Medicina (Kaunas). 2026 Jan 8;62(1):130. doi: 10.3390/medicina62010130 (PMC12843081; doi:10.3390/medicina62010130)
Supplement: Supplementary file 1 [file medicina-62-00130-s001.zip › Supplementary Table S1.pdf]

Study; Year	Age	Race	BMI	ECOG performance status	Comorbidities	Histology	Grade	Stage
Eto; 2013	58	N/A	<20: 32(26%)<br>20 ≤ 25: 57 (45%)<br>≥25: 34 (27%)	0-1: 97 (77%)<br>2-4: 26 (21%)	N/A	Endometrioid: 68 (54%)<br>Non-endometrioid: 57 (46%)	N/A	IVB: 426 (100%)
Holman; 2017	66.3 (46–81)	White: 8 (29,6%)<br>African-American: 13 (48,2%)<br>Hispanic: 4 (14,8%)<br>Asian: 2(7,4%)<br>Other: 0(0%)	29.2 (18.1–68.8)	N/A	N/A	Pure USC: 19 (70,4%)<br>Mixed: 8 (29,6%)	N/A	IIIA: 0 (0%)<br>IIIB: 0 (0%)<br>IIIC1: 0 (0%)<br>IIIC2: 0 (0%)<br>IVA: 2 (7,4%)<br>IVB: 22 (81,5%)
Rajkumar; 2018	<65: 22 (48,9%)<br>≥65: 23 (51,1%)	Caucasian: 32 (71,1%)<br>Other: 13 (28,9%)	N/A	0-1: 43 (95,6%)<br>>1: 2 (4,4%),	Yes: 29 (64,4%)<br>No: 16 (35,5%)	Uterine Papillary Serous Carcinoma (UPSC)/Mixed UPSC: 27 (60%)<br>Endometrioid: 13 (28,9%)<br>Clear cell: 5 (11,1%)	N/A	IIIC: 12 (26,6%)<br>IVA: 1 (2,2%)<br>IVB: 32 (71,1%)
Tobias; 2020	≤40: 57 (4,9%)<br>41-50 : 130 (11,2%)<br>51-60: 415 (35,8%)<br>61-70: 557 (68,1%)	White: 626 (65,8%)<br>Black: 197 (20,1%)<br>Hispanic: 70 (7,4%)<br>Other: 50 (5,3%)<br>Unknown: 0 (0%)	N/A	N/A	N/A	Endometrioid: 321 (27,7%)<br>Serous: 255 (22%)<br>Clear cell: 34 (2,9%)<br>Carcinosarcoma: 176 (15,2%)<br>Sarcoma: 149(12,1%)<br>NOS: 188 (16,2%)<br>Other: 36 (3,1%)	1: 32 (3,4%)<br>2: 94 (9,9%)<br>3: 471 (49,5%)<br>Unknown: 355 (37,3%)	IVA: 122 (10,5%)<br>IVB: 934 (80,6%)<br>IV NOS: 103 (8,9%)
Wright; 2021	65–69: 142 (27,3%)<br>70–74: 189 (27,8%)<br>75–79: 129 (25,5%)	White: 432 (25%)<br>Black: 89 (28,8%)<br>Hispanic: 0 (0%)<br>Unknown or missing: 0(0%)	N/A	0: 336 (25%)<br>1: 134 (25,7%)<br>≥2: 84 (26,6%)	N/A	Endometrioid: 114 (19,3%)<br>Epithelial NOS: 154 (32,9%)<br>Serous: 166 (25,4%)<br>Clear cell: 22 (21%)<br>Carcinosarcoma: 38 (16,9%)<br>Sarcoma: 0 (0%)	1: 17 (23,3%)<br>2: 48 (17,3%)<br>3: 260 (19,4%)<br>Unknown: 229 (46,3%)	IVA: 3037 (100%)

Supplementary Table S1. Chemotherapy Regimens
